# Controlling pH-Dependent Nanozyme Activity by Metal Identity in Histidine-Based Nanoarchitectures

**DOI:** 10.3390/nano16171052

**Published:** 2026-08-24

**Authors:** Zsolt M. Horváth, Árpád Turcsányi, Edit Csapó, Ditta Ungor

**Affiliations:** 1 Department of Physical Chemistry and Materials Science, University of Szeged, Rerrich B. Sq. 1, H-6720 Szeged, Hungary; horvathzsmark@gmail.com (Z.M.H.); juhaszne@chem.u-szeged.hu (E.C.); 2 MTA-SZTE Lendület “Momentum” Noble Metal Nanostructures Research Group, University of Szeged, Rerrich B. Sq. 1, H-6720 Szeged, Hungary; tarpad@chem.u-szeged.hu

**Keywords:** Au, coordination polymer, Cu, nanocluster, fluorescence, peroxidase-like activity, nanozyme, TMB

## Abstract

In this paper, we report a metal-driven structural tuning strategy with the *L*-histidine (His) ligand to modulate peroxidase-like nanozyme performance. Although the His coordinates with gold(III) ions to form an extended helical coordination polymer, its interaction with Cu(II) ions results in quantum-confined, ultrasmall nanoclusters. Based on the optical, structural, and surface analysis, the blue-emitting cores are stabilized by His ligands through imidazole nitrogen coordination, consistent with a Cu-centered coordination environment involved in the observed catalytic activity. According to the catalytic measurements, the His-Cu clusters outperformed the His-Au coordination polymer reference system. Steady-state kinetic modeling and 3D profiling revealed a pronounced shift in the pH optimum: low-valent Cu-containing His-Cu NCs enable efficient peroxide-dependent catalysis under nearly neutral conditions (pH 7.4), whereas His-Au CP exhibits its maximum activity under acidic conditions. Based on these findings, this work highlights nanoscale engineering for tailor-made biomimetic applications.

## 1. Introduction

Artificial enzyme mimics, known as nanozymes, have emerged as an attractive alternative to natural enzymes thanks to their combined catalytic activity with physicochemical stability. Compared to their biological counterparts, nanozymes exhibit improved resistance against temperature and pH variations, prolonged storage stability, lower production costs, and greater compatibility with harsh operating conditions. Since the pioneering discovery of the intrinsic peroxidase-like (POD) activity of Fe_3_O_4_ nanoparticles (NPs) [1], nanozymes have evolved into a broad family of catalytic nanomaterials with enzyme-mimicking activities, including POD, oxidase (Ox), catalase (CAT), superoxide dismutase (SOD), and glutathione peroxidase (GPx) analogs, enabling applications ranging from biosensing and environmental monitoring [2] to biomedicine and catalysis.

Nowadays, among the different classes of nanozymes, noble and transition metal nanoclusters (NCs) have attracted considerable attention owing to their ultrasmall dimensions (<2 nm), molecular-like electronic structures, and exceptionally high surface atom fractions. In contrast to conventional NPs, metal NCs exhibit discrete energy levels due to quantum confinement, enabling the precise modulation of their optical, electronic, and catalytic properties via atomic composition and surface coordination [3]. Their atomically defined metal–ligand interfaces provide unique opportunities to engineer catalytic performance at the molecular level, making metal nanoarchitectures promising candidates for next-generation artificial enzymes [4]. Among enzyme-mimicking reactions, peroxidase-like catalysis remains one of the most extensively investigated because of its central role in oxidation chemistry and its straightforward experimental evaluation using chromogenic substrates such as 3,3′,5,5′-tetramethylbenzidine (TMB) [5]. In metal NC-based systems, hydrogen peroxide (H_2_O_2_) activation generally proceeds through surface-mediated electron transfer, where the oxidation state of surface metal atoms and the local coordination environment determine the formation of reactive oxygen species and, consequently, the overall catalytic efficiency. Considerable efforts have therefore focused on understanding how the structural characteristics influence their POD-like activity [6].

The catalytic regulation provided by surface ligands is particularly important for biomolecule-functionalized nanohybrid systems, where coordination chemistry can generate enzyme-inspired active environments. Among amino acids, histidine (His) represents one of the most multipurpose ligands for construction of biomimetic metal nanostructures due to the strong coordination ability of its imidazole side chain toward transition metal centers [7]. In natural metalloenzymes, His residues frequently participate in the stabilization and regulation of catalytic metal sites by controlling metal coordination geometry, oxidation state transitions, proton transfer pathways, and substrate positioning. Therefore, the incorporation of His into artificial catalytic systems provides a rational strategy for mimicking the functional environment of biologically active sites. His-mediated synthesis has been widely applied for the preparation of fluorescent metal-containing nanoarchitectures, particularly Au and Cu NCs, where the amino acid acts simultaneously as a reducing agent, stabilizing ligand, and functional surface modifier. Fluorescent His-stabilized Au nanoobjects have been extensively investigated due to their excellent fluorescence properties, biocompatibility, and metal–ligand-dependent optical responses [8]. These systems have been used for the detection of metal ions, biomolecules, and other analytes, demonstrating that the histidine coordination environment can provide selective recognition properties while maintaining the unique electronic characteristics of ultrasmall Au NCs [9,10,11,12].

In parallel, His-stabilized Cu NCs have emerged as multifunctional nanomaterials owing to the redox activity of Cu centers and the ability of His to regulate copper coordination [13]. His-Cu NCs have been explored primarily as fluorescent probes for sensing applications, including the detection of metal ions [14,15], small molecules [16,17,18], and biologically relevant analytes [19,20]. The fluorescence response of these systems originates from the unique electronic structure of Cu NCs, where the ligand environment plays a decisive role in controlling cluster formation, stability, and emission properties. These studies established histidine as an effective molecular framework for producing stable and functional Cu-based nanoarchitectures.

The catalytic activity of nanozymes is strongly influenced by environmental parameters, among which pH is one of the most critical factors that determine substrate binding, proton transfer processes, metal oxidation state equilibria, and reactive intermediate formation [5]. For POD-like nanozymes, pH-dependent activity profiles provide important information about catalytic mechanisms because changes in proton availability and surface coordination can directly affect H_2_O_2_ activation and substrate oxidation pathways. Consequently, controlling and understanding pH-responsive catalytic behavior has become an important aspect of designing functional nanozyme systems with predictable activity landscapes [21].

For metal NCs, pH-dependent catalytic behavior is particularly complex because the catalytic process occurs at the dynamic metal–ligand interface, where protonation states of coordinating residues and metal oxidation states can simultaneously influence reaction pathways. In biomolecule-functionalized NCs, amino acid ligands provide additional proton-responsive coordination sites that may regulate accessibility and reactivity of surface metal atoms. Therefore, combining a biologically relevant ligand environment with different metal centers provides an opportunity to investigate how molecular coordination chemistry controls artificial enzyme function.

In our previous work, we established that the interaction of L-histidine with Au(III) precursors at a His:Au molar ratio of 30:1 and pH 6.0 produces a blue-emitting ordered Au(I)-histidine coordination polymer [22]. Structural characterization by mass spectrometry, X-ray diffraction, XPS, and FT-IR indicated an extended helical polymeric architecture in which Au(I) centers are coordinated by the imidazole- and amino-nitrogen donor atoms of histidine. This extended coordination framework differs fundamentally from the quantum-confined, ultrasmall Cu-based architecture investigated here. Such structural differences are expected to influence the accessibility and redox behavior of the metal centers and therefore provide an important structural basis for interpreting the distinct POD-like catalytic responses of the two systems.

Therefore, this work establishes His-based metal nanoarchitectures as a programmable platform for tuning artificial enzyme behavior and provides fundamental insights into the relationship between ligand coordination, metal identity, and catalytic regulation.

## 2. Materials and Methods

### 2.1. Chemicals

*L*-Histidine (His, C_6_H_9_N_3_O_2_, ≥99%); gold(III) chloride trihydrate (HAuCl_4_ × 3 H_2_O, ≥99.9%), copper(II) chloride dihydrate (CuCl_2_ × 2 H_2_O, ≥99.0%), sodium acetate (CH_3_COONa, ≥99.0%), HEPES (C_8_H_18_N_2_O_4_S, ≥99.5%), sodium bicarbonate (NaHCO_3_; ≥99.7%) and 3,3′,5,5′-tetramethylbenzidine (TMB, [-C_6_H_2_(CH_3_)_2_-4-NH_2_]_2_, ≥99%) were purchased from Sigma-Aldrich (Darmstadt, Germany). Acetic acid (CH_3_COOH, 99–100%), sodium hydroxide (NaOH, ≥97.0%), hydrochloric acid (HCl, 37%), dimethyl sulfoxide (DMSO, (CH_3_)_2_SO, ≥99.7%), and sulfuric acid (H_2_SO_4_, 2 M) were products of Molar Chemicals (Budapest, Hungary). All chemicals were used without any further purifications. The fresh stock solutions were prepared with ultrapure Milli-Q water (18.2 MΩ·cm at 25 °C; Merck Millipore, Darmstadt, Germany).

### 2.2. Measurement Techniques

The fluorescent properties of the prepared systems were measured with an ABL&E JASCO FP-8500 spectrofluorometer (ABL&E-JASCO Hungary Ltd., Budapest, Hungary) using a standard quartz cuvette with a 1 cm optical path length. All spectra were registered with 5–5 nm slits, 1 nm resolution, and 500 nm/min scan speed. The absolute internal quantum yield (QY%) was determined using the same instrument with an ABL&E JASCO ILF-835 integrating sphere. The spectra were evaluated in the instrument SpectraManager 2.0 software (ABL&E-JASCO Hungary Ltd., Budapest, Hungary). The fluorescence lifetime was determined using the time-correlated single photon counting (TCSPC) technique on a Horiba DeltaFlex (Horiba Jobin Yvon Ltd., Palaiseau, France) equipped with a DeltaDiode pulse laser (λ_laser_ = 371 nm). The data were collected using 2 nm bandwidth, and the total counts of the peak channel reached 10,000. For the instrument response function (IRF), standard LUDOX^®^ SiO_2_ colloid dispersion (Horiba Jobin Yvon Ltd., Palaiseau, France) was applied. The EzTime software (dedicated to DeltaFlex instrument) from the manufacturer was used to determine the average lifetime and lifetime components. Transmission electron microscopy (TEM) was used to determine the morphology using a Jeol JEM-1400Plus instrument (Jeol Ltd. Tokyo, Japan). The samples were directly dried on a carbon film-coated copper grid. The oxidation state of the copper content, as well as the binding energies of other atoms in the NCs, were investigated by photoelectron spectroscopy (XPS) with a SPECS (SPECS GmbH, Berlin, Germany) instrument equipped with a PHOIBOS 150MCD9 hemispherical analyzer on lyophilized powder samples. The carbon 1s peak (284.80 eV) was used for charge referencing and the Al Kα X-ray source was operated at 200 W power. To discover the possible coordination environment in the His-Cu NCs, the Fourier-transformed Infrared (FT-IR) spectra were recorded using a JASCO FT/IR-4700 instrument with an ATR PRO ONE Single-reflection accessory (ABL&E-JASCO Ltd., Budapest, Hungary). For the evaluation, 256 interferograms were collected with 1 cm^−1^ resolution in the range of 1000 to 4000 cm^−1^. The effect of pH on the size and stability of His-Cu NCs was investigated using dynamic light scattering (DLS) and ζ-potential measurements using a Malvern Zetasizer Nano ZS ZEN 4003 (He-Ne laser with λ = 633 nm, Malvern Panalytical Ltd., Worcestershire, UK) apparatus at room temperature. The POD-like activity was investigated using UV-Vis spectrophotometry on an ABL&E JASCO V-770 type spectrophotometer (ABL&E-JASCO Hungary Ltd., Budapest, Hungary) equipped with an ABL&E-JASCO ETCS-761 Peltier-based temperature-controlled sample holder. Steady-state catalytic activity was evaluated through absorbance measurements at 450 nm, while 652 nm was selected for the kinetic studies at 20.0 ± 0.1 °C. The samples were inserted into the instrument in 1 cm standard quartz cuvettes and the data were evaluated in the SpectraManager 2.5 software of the ABL&E JASCO manufacturer.

### 2.3. Synthesis of His-Au(I) CPs and His-Cu NCs

The blue-fluorescent His-Au CP was prepared based on our previously published work [22], where the applied molar ratio was His:Au/30:1 and the total gold concentration was 1 mM. The synthesis was carried out at pH 6.0 and the prepared system was purified with centrifugation at 13,000 rpm.

For the synthesis of His-Cu NCs, 10 mL of 0.3 M His solution was diluted with 3.5 mL MQ-water, and the pH was adjusted to pH 10.0 by 1 M NaOH solution based on the addition of 1.62 mL CuCl_2_ solution (0.01 M). After mixing, a further 5 min of magnetic stirring was performed. For nucleation and cluster growth, the sample was thermostated at 80 °C for 24 h. The final product was purified by dialysis in a Spectra/Por^®^ Float-A-Lyzer^®^ G2 dialysis device (cut-off: 100–500 Da; VWR International Ltd., Debrecen, Hungary). The synthesis parameters were experimentally optimized by systematically varying the His:Cu molar ratio, Cu(II) concentration, pH, reaction temperature, and reaction time, while monitoring the resulting fluorescence response of the products. For each parameter, the condition providing the maximum fluorescence response within the investigated range was selected (Figure S1). The optimized synthesis conditions were a His:Cu/250:1 molar ratio, pH 10.0, 80 °C, and reaction duration of 24 h, without the addition of an external reducing agent. After purification, the final His:Cu molar ratio was experimentally determined to be approximately 225:1, based on the His content measured by UV–Vis spectrophotometry and the Cu content determined by ICP-MS (the Cu content was determined by ICP-MS using an Agilent 7700x quadrupole ICP-MS instrument (Agilent Technologies, Santa Clara, CA 95051, USA) at the University of Szeged, Szeged, Hungary).

### 2.4. Determination of Possible Peroxidase-Like Catalytic Behaviors

To measure the possible nanozyme properties of both systems, the peroxidase-like activity was investigated. During these measurements, the model reaction of the H_2_O_2_-to-water conversion was selected in the presence of TMB as chromogenic dye. For this purpose, two methods were selected. In the first case, a stationary measurement using the stop-bath method was performed, where the optimal catalytic pH and metal concentration were examined. In practice, the pH was regulated by acetic acid/acetate (pH 3.0 and 4.5), HEPES (pH 7.4), and sodium bicarbonate (pH 10.1) buffer solutions at 50 mM concentration. The final metal content varied between 3 and 750 µM in individual samples. To monitor the color changes, 40 µL TMB solution (10 mg mL^−1^) was mixed into the bottle. The enzymatic reaction was initiated by adding 155 µL H_2_O_2_ from 0.3 *v*/*v*% solution. After 10 min, the reaction was stopped by adding 2 mL 0.1 M H_2_SO_4_. For the evaluation, the absorbance changes (Δ*A*) were calculated after correction of blank samples. The absorbances were detected at 450 nm and the blank correction was completed at the appropriate buffer solutions. The Δ*A* values were converted into oxidized TMB concentration based on Equation (1):

(1) $$c_{oxTMB}\;\left(\mu M\right)=\;\frac{∆A}{\varepsilon \times l}$$

where Δ*A* is the absorbance change at 450 nm, *ε* is the molar absorption coefficient of the oxidized TMB molecule (59,000 M^−1^ cm^−1^), and l is the optical length (1 cm).

Secondly, a kinetic method was selected using the optimal catalytic conditions identified by the stop-bath measurements. Specifically, pH 4.5 and a gold concentration of 750 µM were applied for His-Au CP, whereas a Cu concentration of 500 µM and near-neutral conditions (pH 7.4) provided the highest POD-like activity for His-Cu NCs. In the individual samples, the concentration of H_2_O_2_ was varied between 5 and 100 mM. The absorbance change was monitored at 647 nm based on the blue color of the TMB charge-transfer complex formation at 20.0 ± 0.1 °C. To determine the apparent kinetic parameters (maximal velocity/*v_max_*, half-maximal concentration parameter/*K* and Hill coefficient/*n*), the kinetic curves were fitted using the Hill model [23,24].

## 3. Results and Discussion

### 3.1. Histidine-Directed Formation of Au and Cu Metal Architectures

**Figure 1:**
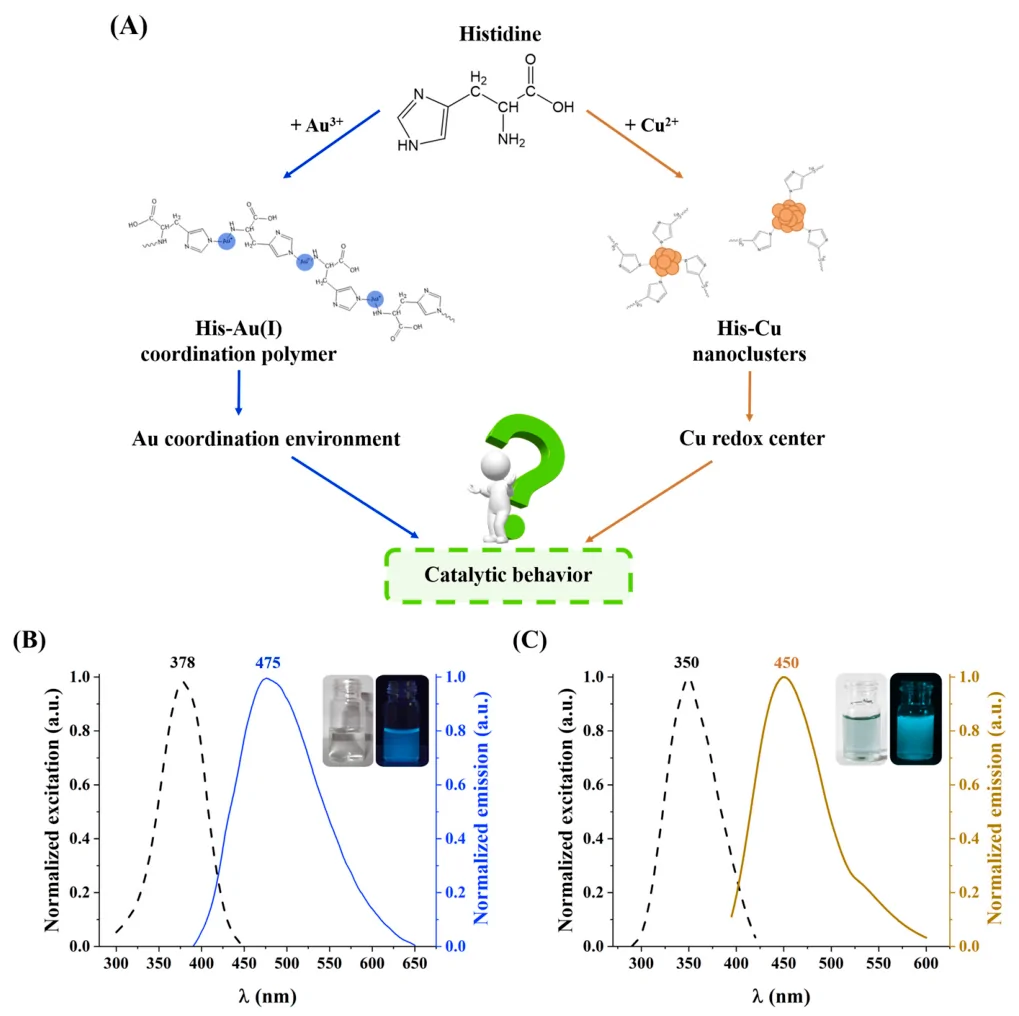
(**A**) Schematic illustration of the concept of metal-dependent catalytic programming using histidine as a common coordination ligand. (**B**,**C**) Fluorescence excitation (dashed line) and emission (continuous line) spectra of histidine-stabilized Au coordination polymer and Cu nanoclusters, respectively, with photos of samples in daylight and under UV lamp (λ = 365 nm).

The core conceptual framework of this study is systematically illustrated in Figure 1A, highlighting the metal-driven structural and catalytic relationship within the same ligand environment. In our previous work, the interaction between His and precursor Au(III) ions was demonstrated to form a well-defined His-Au CP. This His-Au CP showed intensive blue emission, as presented in Figure 1B (λ_ex_/λ_em_ = 378/475 nm). The structure was characterized by XPS, mass spectrometry, X-ray diffraction, and FT-IR spectroscopy, clearly demonstrating the ordered helical polymeric structure with the presence of Au(I) active centers and with the N_imidazole_- and N_amino_-donor atom attached to its backbone [22]. Expanding on this coordination strategy, this work focuses on the direct reduction of Cu(II) ions in the presence of the same His ligand. The main goal of this comparative study was to examine how the change in the coordinated metal from Au to Cu within a histidine-based ligand framework is associated with changes in the resulting metal–ligand architecture and POD-like catalytic behavior.

After the synthesis and purification, the His-Cu sample shows blue emission under a UV lamp, while the aqueous solution appeared transparent and slight greenish shade in daylight. The characteristic plasmon band of copper nanoparticles between 560 and 590 nm does not appear on the UV-Vis spectrum (Figure S2A). The prepared His-Cu system shows excitation and emission maxima at 350 and 450 nm, respectively. The determined QY% is 3.5 ± 0.3%. The fluorescence decay profile (Figure S2B) was fitted by a tri-exponential function (χ^2^ = 1.05). Based on the fitting, three main fluorescence lifetime components can be separated: *τ*_1_: 0.21 ± 0.01 ns (19.4%); *τ*_2_: 1.45 ± 0.12 ns (36.2%); and *τ*_3_: 4.60 ± 0.13 ns (44.4%). The fastest *τ*_1_ component can be attributed to the non-radiative relaxation processes on the cluster surface. The *τ*_2_ component perhaps belongs to delocalized states between the surface ligand–Cu cores. The *τ*_3_ is the largest component, which can be attributed to the ligand-to-metal charge-transfer (LMCT) processes, showing the strong electronic coupling between His and Cu [25].

Based on the optical characteristics, including the large Stokes shift and multicomponent fluorescence decay, the observed emission is consistent with the formation of few-atom, quantum-confined Cu nanoclusters [13,25,26]. Although the Jellium model has been successfully applied to correlate the electronic structure and optical properties of metal nanoclusters with their size [27], its applicability to ligand-protected Cu nanoclusters is limited because metal–ligand interactions and the local coordination environment can substantially influence electronic structure and emission properties [28]. In particular, structurally characterized histidine-protected Cu nanoclusters with different Cu nuclearities have been reported to exhibit similar emission maxima, demonstrating that the emission wavelength alone cannot be used as a reliable measure of cluster nuclearity HIV. Therefore, the observed emission maximum at 450 nm is considered here as evidence supporting the formation of a quantum-confined Cu nanocluster system, rather than as a reliable means of assigning an exact number of Cu atoms. Precise determination of the cluster nuclearity would require direct structural or compositional characterization.

**Figure 2:**
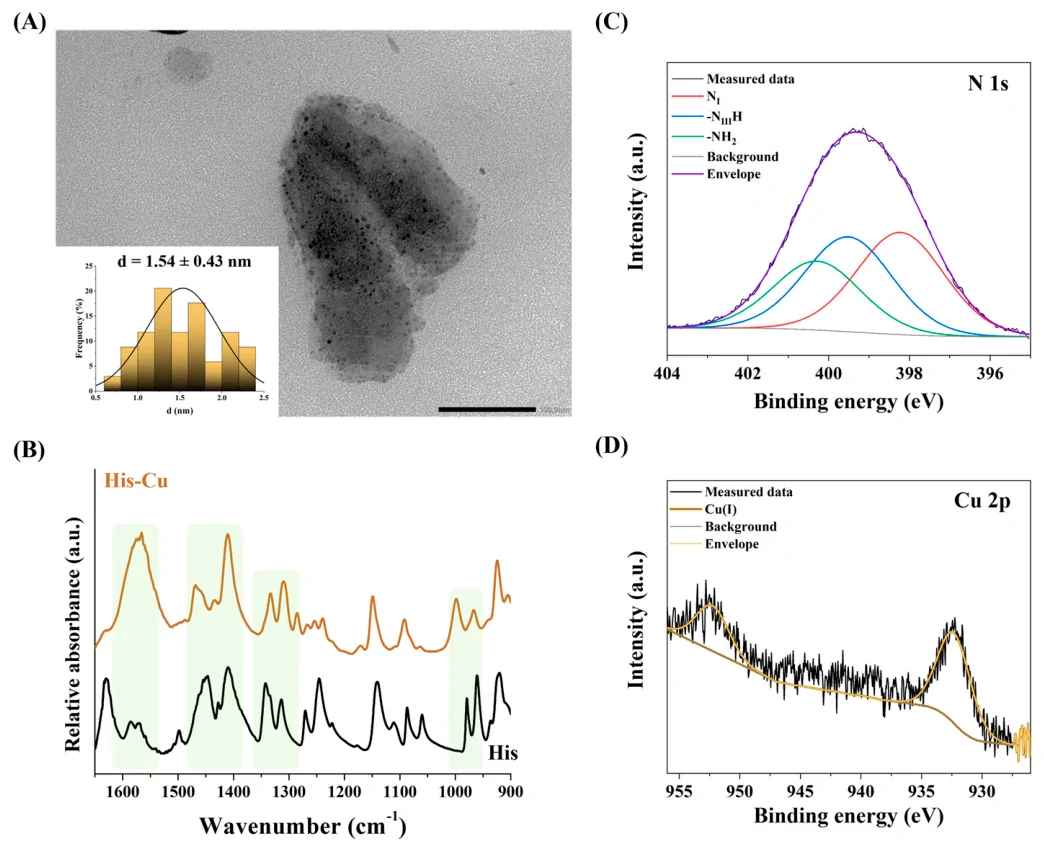
(**A**) Representative TEM image showing the nanoscale morphology of dried His-Cu NC sample with the corresponding size distribution analysis. (**B**) The FT-IR spectrum of pure His (black) and His-Cu NCs (brown). (**C**,**D**) The XPS spectra of the N 1s and Cu 2p region, respectively.

The morphology and size distribution of the dehydrated His-Cu sample were investigated by TEM. As shown in Figure 2A, the TEM image reveals dark spherical dots corresponding to the copper-containing cores. These cores have an average diameter of 1.54 ± 0.43 nm and are uniformly embedded within a larger, light-contrasted organic His matrix, creating a complex architecture.

To firmly establish the coordination mechanism between His-Cu, FT-IR spectroscopy was performed, comparing the spectra of pure His and as-synthesized His-Cu NCs (Figure 2B). The FT-IR spectrum of the NCs exhibits profound alterations and characteristic shifts in the fingerprint region (marked by green). The broad and intense peak observed around 1570–1590 cm^−1^ is attributed to the combined contributions of the asymmetric carboxylate stretching (ν_as_(COO¯)) and the (C=N/C=C) skeletal vibrations of the imidazole ring, which are significantly altered by metal coordination. Crucially, the vibrational modes associated with the imidazole ring dynamics, including the in-plane ring deformations at ~1460 cm^−1^ and 1300–1340 cm^−1^, as well as the out-of-plane bending modes at 950–1000 cm^−1^, all display distinct blue shifts towards higher wavenumbers after cluster formation [29,30]. These spectral changes provide strong evidence that the His molecules are coordinated to the Cu-containing core primarily through the imidazole nitrogen atoms.

This coordination mode assignment is further supported by the high-resolution N 1s XPS data (Figure 2C). This spectrum was deconvoluted into three distinct peaks assigned to imine nitrogen (N_I_ at 398.2 eV), amine/pyrrole-type nitrogen (–N_III_H at 399.5 eV), and free amino backbone groups (–NH_2_ at 400.3 eV). The prominent chemical shifts in the imidazole nitrogen peaks demonstrate that His ligands are coordinated to the Cu-containing core primarily via the imidazole ring, while amino groups remain comparatively less affected, consistent with their preservation in the ligand shell [31]. In addition to the coordination mechanism, the surface chemical composition was also investigated by XPS. The Cu 2p spectrum (Figure 2C) exhibits the characteristic spin–orbit doublet of 2p_3/2_ and 2p_1/2_ at 932.5 and 952.4 eV, respectively. The asymmetric deconvolution and the absence of shake-up satellite features in the 940–945 eV region is consistent with the predominantly low-valent state of Cu species. The detected binding energies are in good agreement with the data in the previously published literature [32], which has reported reduced cluster cores with stable mixed valence Cu^+^/Cu^0^ domains. Based on further investigation, the C 1s spectrum (Figure S3A) clearly shows the intact amino acid framework. The O 1s region (Figure S3B) refers to a high density of surface hydroxide groups with adsorbed H_2_O, which can facilitate substrate diffusion and fast electron transfer pathways during enzymatic measurements.

To assess the colloid stability, surface-charge dynamics, and photoluminescent property of His-Cu NCs under varying environments, pH-dependent DLS, ζ-potential, and PL measurements were performed (Figure S3). In the pH range of 4.0 to 12.0, the NCs exhibited excellent colloidal stability, maintaining a well-dispersed small hydrodynamic size. This stability was supported by the increasingly negative zeta-potential (ca. −17 mV), which originates from the deprotonation of carboxyl groups in the His shell. In contrast, a dramatic structural and optical transition was captured in the highly acidic region (pH 1.0–3.0). At pH 3.0, according to the isoelectric point—where the net surface charge drops near 0 mV—electrostatic repulsion vanishes, triggering the controlled assembly of NCs into large macro-aggregates with a hydrodynamic diameter of about 400–500 nm. Simultaneously, the PL intensity profile reaches its absolute maximum at pH 2.0. This sharp luminescence enhancement is a hallmark of Aggregation-Induced Emission (AIE), driven by the protonation-induced restriction of intramolecular motions within the rigidified His scaffold.

### 3.2. Metal-Dependent Peroxidase-Like Catalytic Activity

As mentioned above, for comparison of the possible POD-like activity, a His-Au CP was also employed as a reference system. While the His-Cu system forms quantum-confined NCs with active Cu(I) redox centers, the His-Au system forms an extended, ordered helical coordination polymer containing Au(I) centers coordinated by His through imidazole- and amino-nitrogen donor atoms [22]. This polymeric architecture provides a structurally distinct coordination environment compared with the ultrasmall His-Cu NCs.

**Figure 3:**
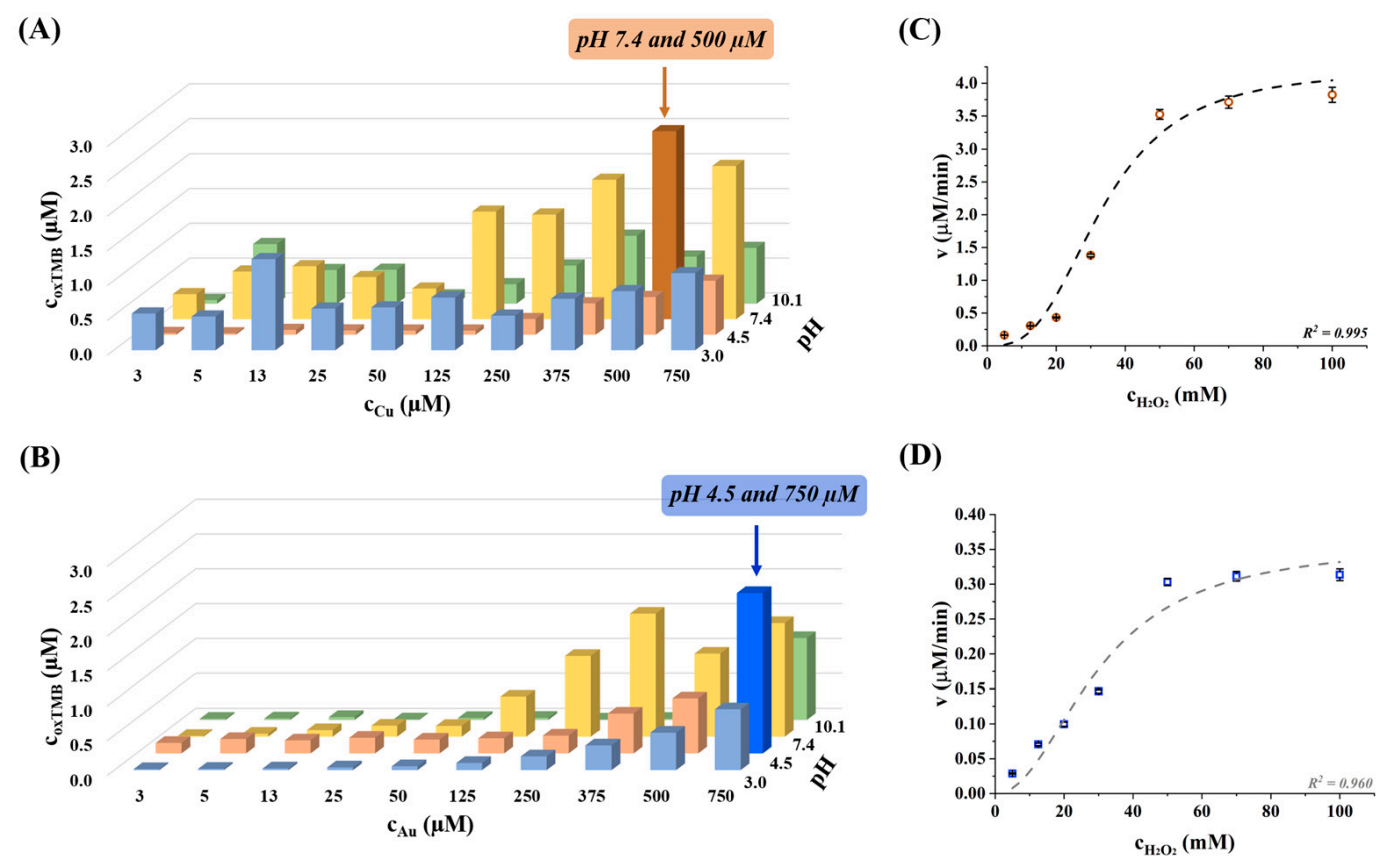
(**A**,**B**) Effect of the pH and metal content on the oxidized TMB concentration determined by TMB/H_2_O_2_ assay via “stop-bath” method. (**C**,**D**) The H_2_O_2_-dependent initial velocity profile of His-Cu NCs and His-Au CP, respectively, fitted by Hill kinetic model.

The POD-like nanozyme activity of the His-derived fluorescent nanomaterials was systematically evaluated using a stop-bath colorimetric assay across a wide range of Cu and Au concentrations (0–750 μM) and four pH values (3.0–10.0). The 3D profiling reveals that the catalytic efficiency is highly dependent on both the pH environment and the metal concentration. The difference in structural organization is reflected in the catalytic responses. The His-Cu NCs exhibited outstanding performance at near-neutral pH (pH 7.4) (Figure 3A), with the highest responses recorded at 500 µM metal content. The existence of a distinct concentration optimum suggests that excessive Cu loading does not proportionally enhance catalytic performance, indicating that the accessibility or efficiency of catalytically active sites may become limited at higher metal concentrations.

In contrast, the POD-activity of Au CP (Figure 3B) was strongly favored under acidic conditions. The maximum catalytic response was reached at pH 4.5 and 750 µM metal content. These findings demonstrate that replacing the metal center in the His coordination framework shifts the optimal catalytic window toward acidic conditions, highlighting the important role of the coordinated metal in regulating nanozyme activity.

To investigate the peroxide-dependent catalytic behavior, the formation of the oxidized blue charge-transfer TMB complex was monitored continuously in individual samples, where the concentration of H_2_O_2_ was varied between 5 and 100 mM. Representative kinetic traces are shown in Figure S5 and the corresponding initial reaction rates were used for Hill model fitting.

The peroxide-dependent catalytic activity of His-Cu NCs followed a sigmoidal profile (Figure 3C) and was well described by the Hill model (R^2^ = 0.995). The Hill coefficient was greater than unity (*n* = 2.94 ± 0.83), indicating a pronounced non-linear response to increasing H_2_O_2_ concentration. In classical enzymatic systems, *n* > 1 is commonly associated with positive cooperativity; however, for heterogeneous nanozyme systems, a Hill coefficient above unity does not by itself establish cooperative substrate binding. The observed behavior may instead reflect a combination of interacting or heterogeneous catalytic sites, the dual role of H_2_O_2_ as a reactant and oxidant in the POD-like reaction, and/or coupled redox activation steps at the metal–ligand interface. Therefore, the Hill coefficient is interpreted here as an empirical descriptor of the sigmoidal peroxide-dependent response rather than as direct evidence of a specific cooperative mechanism.

The His-Au CP also exhibited Hill-type peroxide-dependent behavior (R^2^ = 0.960), with n = 2.12 ± 0.71, indicating that a similar non-linear H_2_O_2_ response is present in both His-coordinated systems. Importantly, the apparent half-maximal concentration parameters were comparable for His-Cu NCs (K = 33.35 ± 7.16 mM) and His-Au CP (K = 30.11 ± 6.65 mM), whereas the apparent maximum velocity was substantially higher for His-Cu NCs (4.20 ± 0.63 µM min^−1^) than for His-Au CP (0.36 ± 0.05 µM min^−1^). These results suggest that the principal difference between the two systems lies in their catalytic capacity and pH-dependent activity rather than in their apparent H_2_O_2_ concentration dependence. All determined apparent kinetic parameters can be found in Table 1.

Collectively, these results demonstrate that while both His-coordinated metal systems exhibit similar peroxide-responsive kinetic behavior, the coordinated metal ion contributes substantially to defining the optimal catalytic pH window and the overall catalytic efficiency of the POD-like reaction [33].

### 3.3. Proposed Mechanism for Metal-Dependent Nanozyme Regulation

The experimental results consistently demonstrate that the His serves as an effective coordination scaffold for both Cu- and Au-based nanoarchitectures, while the coordinated metal center dictates the catalytic behavior. Despite sharing the same ligand environment, His-Cu NCs and His-Au CP exhibit markedly different optimal catalytic conditions and catalytic capacities, indicating that the coordinated metal is an important factor in defining the catalytic response. The pH-dependent activity profiles revealed distinct catalytic windows for the two materials. His-Cu NCs reached their maximum POD-like activity at near-neutral pH (7.4), while His-Au CP showed its highest activity under mildly acidic conditions (pH 4.5). These observations suggest that the electronic structure and redox characteristics of the coordinated metal govern the peroxide activation process and determine the reaction conditions under which catalytically active species are most efficiently generated.

To further compare the intrinsic catalytic performance of the two systems, the maximum catalytic rates were normalized to the total metal concentration under the respective optimized reaction conditions [34]. The apparent turnover frequencies (*TOF_app_*) were calculated using Equation (2):

(2) $${TOF}_{app}=\;\frac{v_{max}}{\left[M\right]}$$

where *v_max_* is the maximal oxTMB formation velocity (µM min^−1^) and [*M*] is the total metal concentration (µM) in the reaction mixture.

The resulting *TOF_app_* values were 8.4 × 10^−3^ min^−1^ for Cu NCs and 4.8 × 10^−4^ min^−1^ for His-Au CP, indicating approximately 17.5-fold higher metal-normalized catalytic activity for the Cu-based nanozyme. Although these values do not represent true enzymatic turnover numbers, as the exact number of catalytically active metal sites is unknown, they provide a useful comparative metric for evaluating the intrinsic catalytic efficiency of the two histidine-coordinated systems.

The substantially higher apparent turnover frequency of His-Cu NCs indicates a markedly higher metal-normalized catalytic efficiency under the respective optimized conditions. However, because the two systems differ not only in metal identity but also in nuclearity, coordination environment, and overall architecture, the individual contribution of these factors cannot be separated based on the present comparison. To further examine the possible redox changes occurring during peroxide activation, additional UV–Vis measurements were performed. Upon addition of H_2_O_2_, the original His-Cu NC spectrum changed and a broad absorption feature appeared in the blue spectral region (Figure S5). Similar spectral changes have been reported for Cu(II)-histidine coordination systems, although the exact spectral position and intensity depend strongly on the coordination environment, pH, and ligand-to-metal ratio [35,36,37]. Therefore, the observed spectral change is consistent with an alteration of the copper oxidation and/or coordination state upon H_2_O_2_ treatment, but the UV–Vis data alone do not allow unambiguous assignment of a specific His-Cu(II) coordination structure. In the context of the observed POD-like activity and the low-valent Cu species identified by XPS, the formation of transient oxidized Cu species during H_2_O_2_ activation is therefore considered a plausible mechanistic pathway.

**Figure 4:**
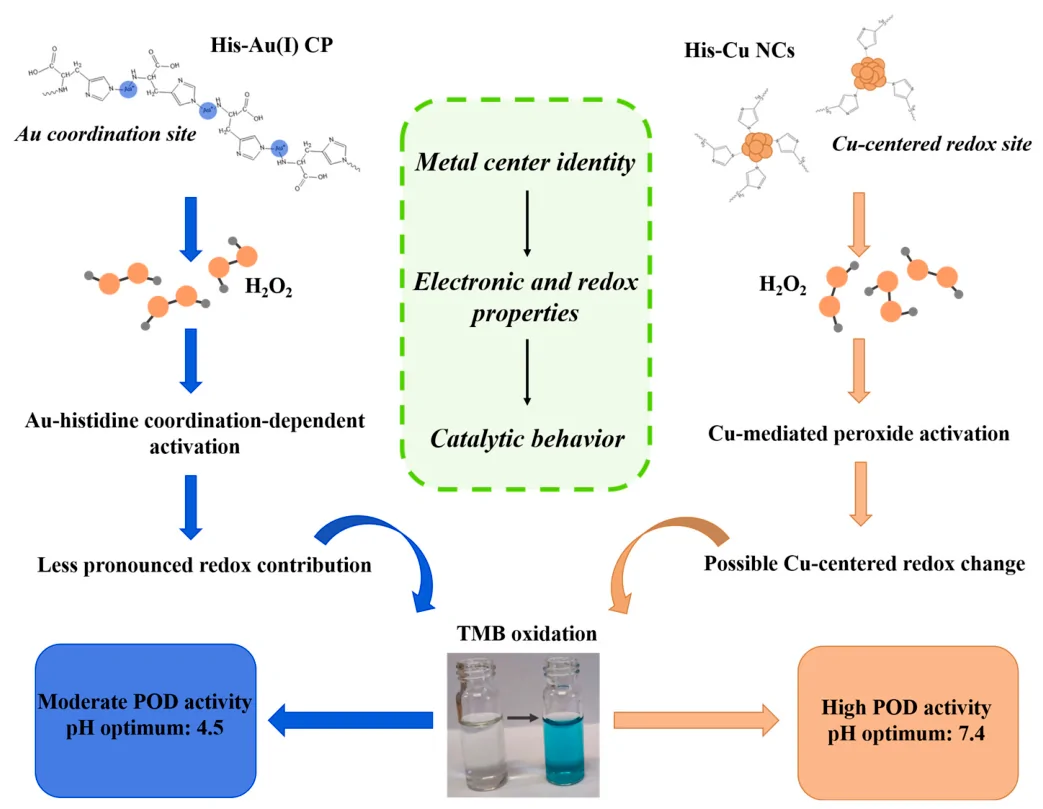
Proposed mechanism for the metal-dependent modulation of POD-like activity in histidine-coordinated metal systems.

Based on these observations, a mechanistic model is proposed (Figure 4), in which histidine functions as a common coordination scaffold, whereas the coordinated metal programs the catalytic pH window, the apparent turnover efficiency, and the overall POD-like activity.

## 4. Conclusions and Future Perspective

In this paper, we have comprehensively characterized the physicochemical properties of the synthesized His-Cu NCs. Through the systematic combination of optical and structural characterization techniques, it was established that His-derived ligands coordinate to the ultrasmall Cu-based nanostructure, with XPS supporting the presence of low-valent Cu species and FT-IR indicating the involvement of the imidazole functionality in Cu coordination. The POD-like catalytic activity was subsequently investigated and compared with that of our previously synthesized His-Au CP.

Our comparative assessment demonstrates that the change in coordinated metal within a His-based ligand framework is associated with pronounced differences in nanoscale architecture, coordination environment, and POD-like catalytic behavior. Although the present comparison does not allow the individual contributions of metal identity, morphology, and coordination environment to be fully separated, the substantially higher metal-normalized apparent catalytic activity of His-Cu NCs highlights the importance of the resulting metal–ligand structural and electronic environment in the observed catalytic response. The transition from the extended Au polymer architecture to the quantum-confined Cu NC architecture was associated with an approximately 17.5-fold higher metal-normalized apparent catalytic activity for the His-Cu NCs.

Furthermore, the two systems exhibited distinct pH-dependent catalytic profiles, with His-Cu NCs showing maximum POD-like activity at near-neutral pH, whereas the His-Au CP exhibited its highest activity under acidic conditions. The kinetic analysis revealed comparable apparent half-maximal concentration parameters for H_2_O_2_, while His-Cu NCs exhibited a substantially higher apparent maximum velocity. These findings indicate that the two histidine-coordinated architectures possess markedly different catalytic efficiencies and pH-dependent responses. The observed peroxide-dependent spectral changes in the His-Cu system are consistent with alterations in the copper oxidation and/or coordination state during H_2_O_2_ activation; however, the available data do not allow assignment of a specific transient Cu(II) coordination structure.

Overall, these results demonstrate that histidine provides a versatile coordination framework within which different metal centers can generate distinct nanoscale architectures and catalytic behaviors. Rather than attributing the observed differences exclusively to metal identity, our results highlight the coupled influence of the coordinated metal and the resulting metal–ligand architecture on the catalytic properties of histidine-based nanozymes. This comparative approach provides a useful basis for the rational development of amino-acid-coordinated metal nanostructures with tunable catalytic properties.

## Figures and Tables

**Table 1:** Apparent kinetic parameters (*v_max_*, *K*, and *n*) from the fitting of the Hill kinetic model of H_2_O_2_-dependent POD-like catalytic activity.

System	Model	*v_max_* (µM min^−1^)	*K* (mM H_2_O_2_)	*n*	R^2^
His-Cu NCs	Hill	4.20 ± 0.63	33.35 ± 7.16	2.94 ± 0.83	0.995
His-Au CP	Hill	0.36 ± 0.05	30.11 ± 6.65	2.12 ± 0.71	0.960

## Data Availability

The original contributions presented in this study are included in the article/supplementary material. Further inquiries can be directed to the corresponding author.

## Supplementary Materials

The following supporting information can be downloaded at https://www.mdpi.com/article/10.3390/nano16171052/s1: Figure S1: Optimization of the synthesis conditions of His-Cu NCs. The effects of (A) His:Cu molar ratio, (B) Cu(II) concentration, (C) pH, (D) reaction temperature, and (E) reaction time on the fluorescence intensity of the resulting His-Cu NCs were systematically investigated. Figure S2: (A) The UV-Vis spectra of freshly prepared His-Cu NCs. (B) Typical fluorescence decay curve of His-Cu NCs with the instrument response function (IRF, gray dots) and fitting. Figure S3: The XPS spectra of (A) C 1s and (B) O 1s. Figure S4: (A) The hydrodynamic diameters (d_H_) with the ζ-potential values, and (B) PL maxima at 450 nm depending on the pH. Figure S5: Typical primer kinetic curves of (A) His-Cu NCs and (B) His-Au CP depending on the H_2_O_2_ concentration. Figure S6: The UV-Vis spectra of His-Cu NCs after H_2_O_2_ treatment at 50 mM concentration.
