# Supplementary material for: Controlling pH-Dependent Nanozyme Activity by Metal Identity in Histidine-Based Nanoarchitectures
Source: Nanomaterials (Basel). 2026 Aug 24;16(17):1052. doi: 10.3390/nano16171052 (PMC13567170; doi:10.3390/nano16171052)
Supplement: Supplementary file 1 [file nanomaterials-16-01052-s001.zip › nanomaterials-4505821-supplementary.pdf]

SUPPORTING INFORMATION

Control the pH-Dependent Nanozyme Activity by Metal Identity in Histidine-Based Nanoarchitectures

Zsolt M. Horváth [2], Árpád Turcsányi [1], Edit Csapó [1, 2] and Ditta Ungor [2, *]

[1] MTA-SZTE Lendület "Momentum" Noble Metal Nanostructures Research Group, University of Szeged, Rerrich B. Sq. 1, H-6720 Szeged, Hungary

[2] Department of Physical Chemistry and Materials Science, University of Szeged, Rerrich B. Sq. 1, H-6720 Szeged, Hungary

* Correspondence: D. Ungor, ungord@chem.u-szeged.hu

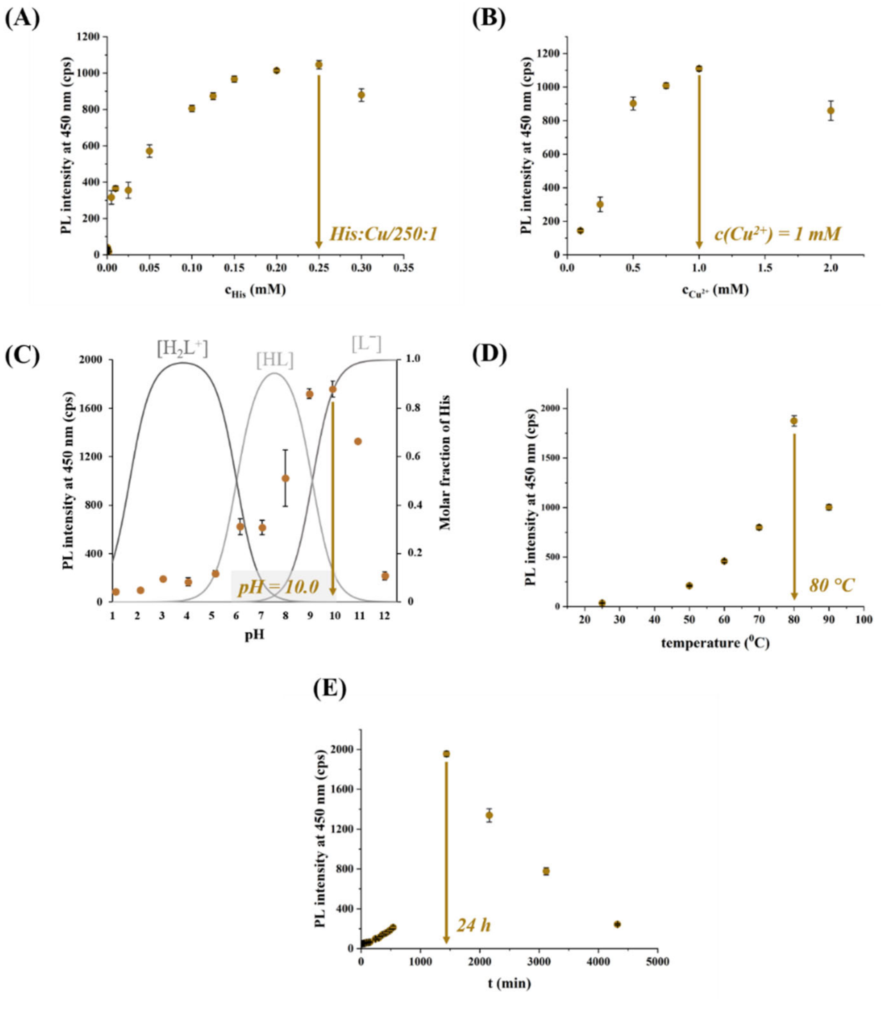

Figure S1: Optimization of the synthesis conditions of His-Cu NCs. The effects of **(A)** His:Cu molar ratio, **(B)** Cu(II) concentration, **(C)** pH, **(D)** reaction temperature, and **(E)** reaction time on the fluorescence intensity of the resulting His-Cu NCs were systematically investigated.

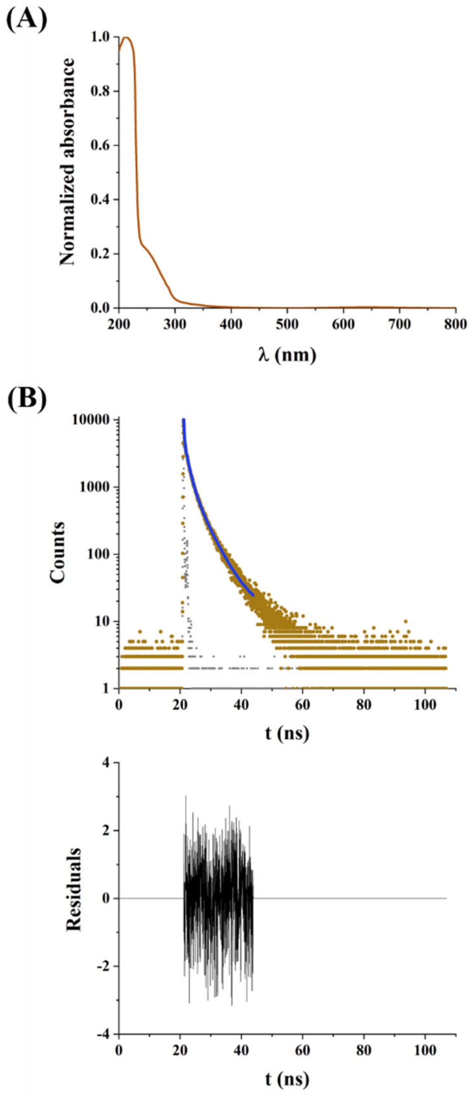

Figure S2: (A) The UV-Vis spectra of freshly prepared His-Cu NCs. **(B)** Typical fluorescence decay curve of His-Cu NCs with the instrument response function (IRF, gray dots) and fitting.

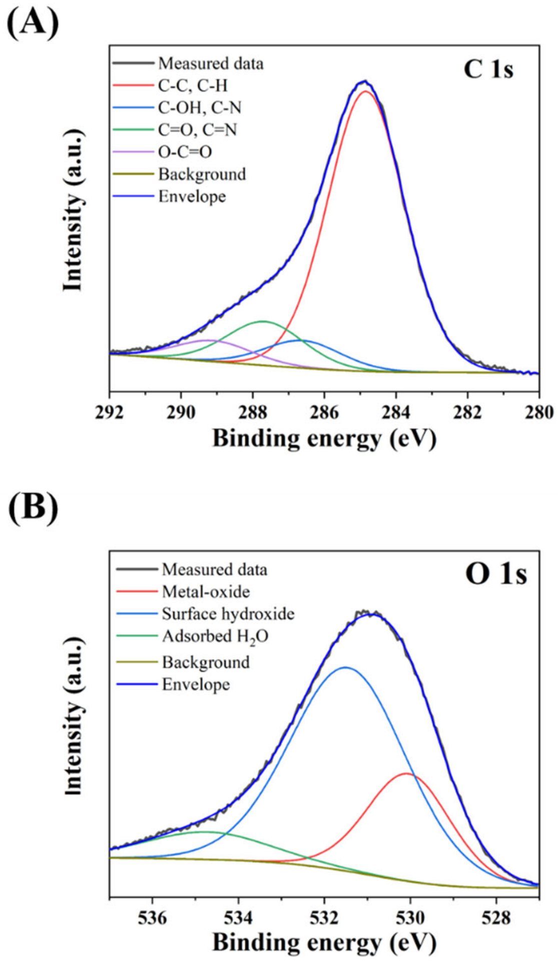

Figure S3: The XPS spectra of **(A)** C 1s and **(B)** O 1s.

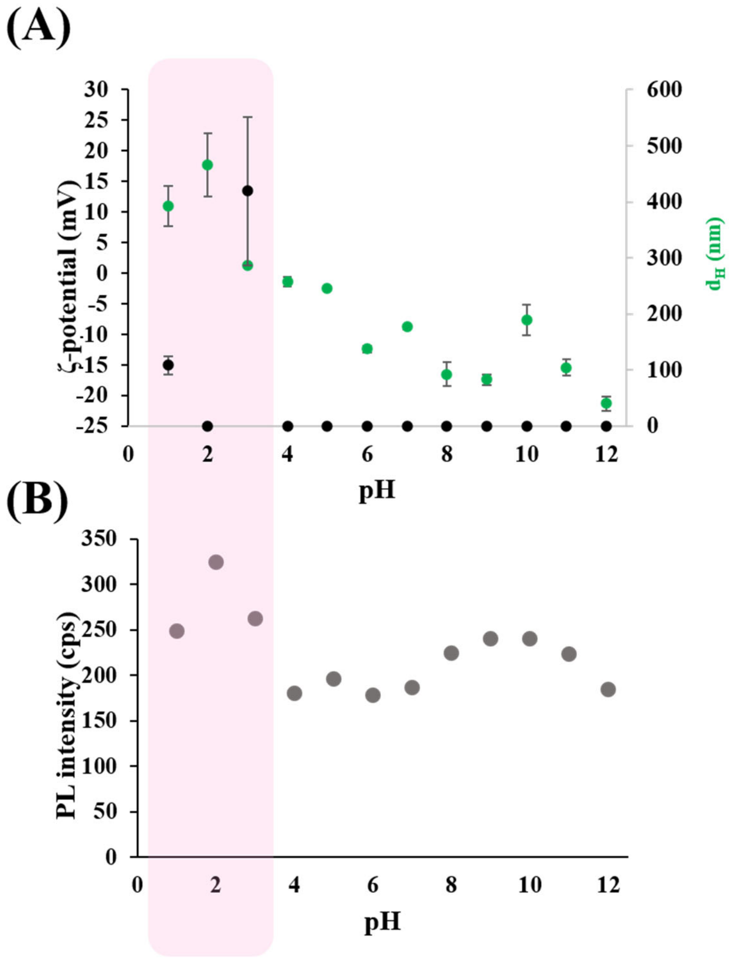

Figure S4: (A) The hydrodynamic diameters (d_H) with the ζ-potential values, and **(B)** PL maxima at 450 nm depending on the pH.

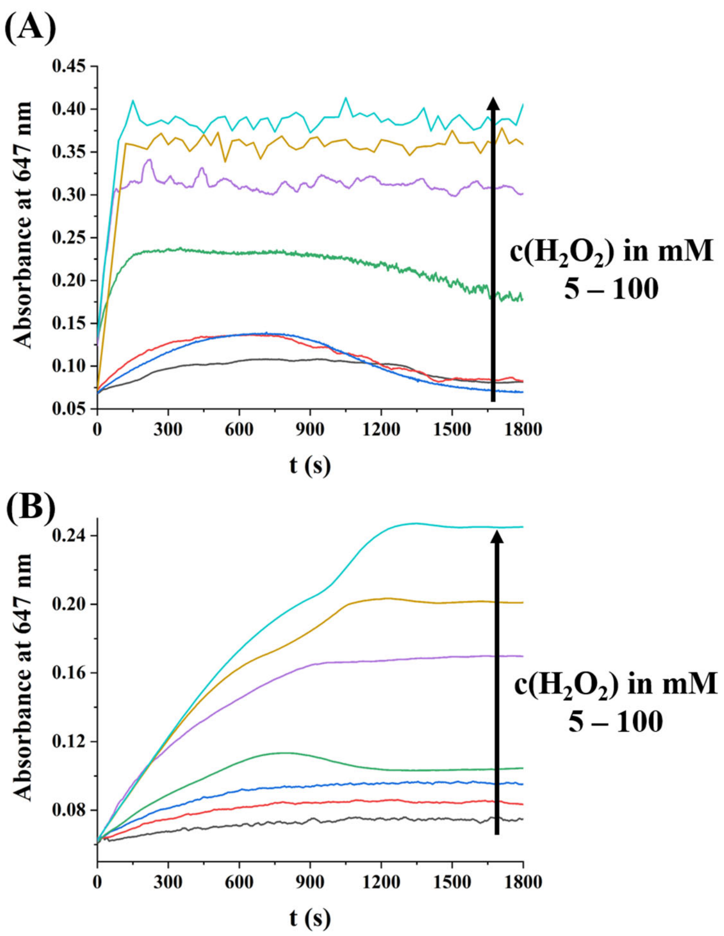

Figure S5: Typical primer kinetic curves of **(A)** His-Cu NCs and **(B)** His-Au CP depending on the H_2O_2 concentration.

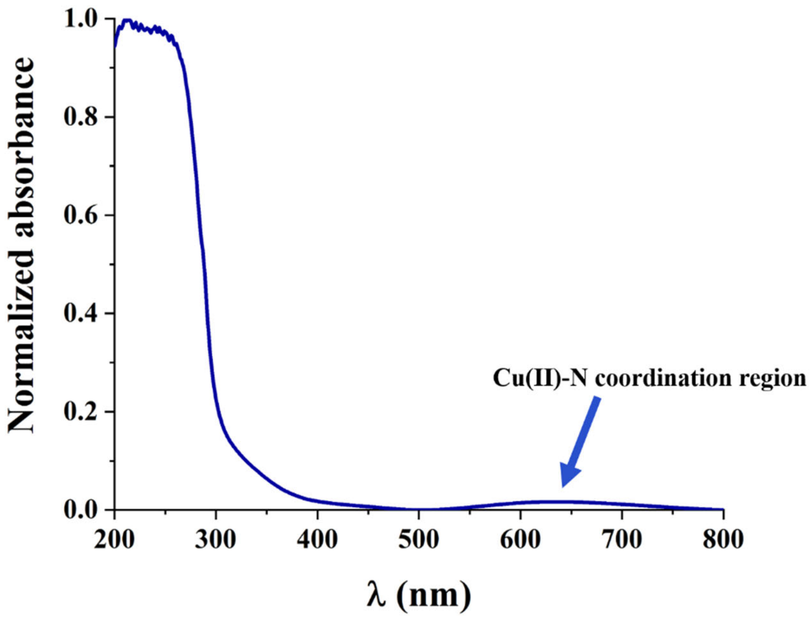

Figure S6: The UV-Vis spectra of His-Cu NCs after H_2O_2 treatment at 50 mM concentration.
